# Separating Geometric and Diffusive Contributions to the Surface Nucleation of Dislocations in Nanoparticles

**DOI:** 10.1021/acsnano.3c09026

**Published:** 2024-01-26

**Authors:** Ruikang Ding, Soodabeh Azadehranjbar, Ingrid M. Padilla Espinosa, Ashlie Martini, Tevis D. B. Jacobs

**Affiliations:** † Department of Mechanical Engineering and Materials Science, University of Pittsburgh, Pittsburgh, Pennsylvania 15261, United States; ‡ Department of Mechanical Engineering, University of California, Merced, Merced, California 95340, United States

**Keywords:** *In Situ* TEM, Mechanical Behavior, Platinum Nanoparticles, Surface Dislocation Nucleation, Surface Diffusion, Surface Termination

## Abstract

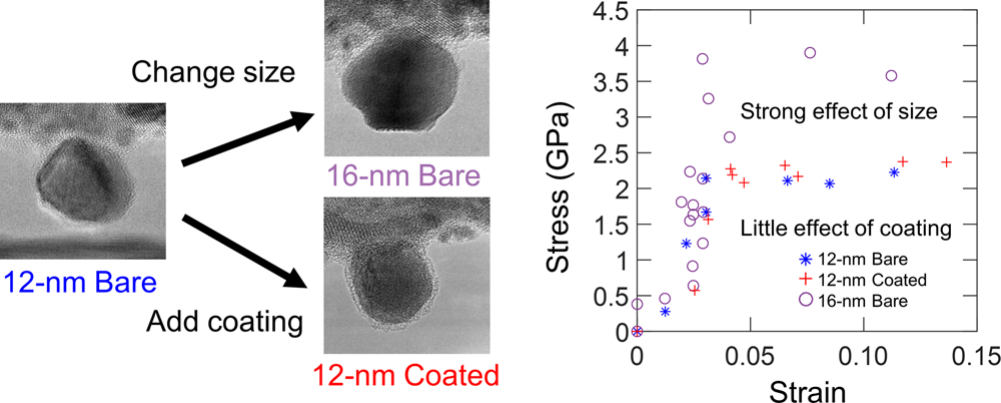

While metal nanoparticles are widely used, their small size makes them mechanically unstable. Extensive prior research has demonstrated that nanoparticles with sizes in the range of 10–50 nm fail by the surface nucleation of dislocations, which is a thermally activated process. Two different contributions have been suggested to cause the weakening of smaller particles: first, geometric effects such as increased surface curvature reduce the barrier for dislocation nucleation; second, surface diffusion happens faster on smaller particles, thus accelerating the formation of surface kinks which nucleate dislocations. These two factors are difficult to disentangle. Here we use *in situ* compression testing inside a transmission electron microscope to measure the strength and deformation behavior of platinum particles in three groups: 12 nm bare particles, 16 nm bare particles, and 12 nm silica-coated particles. Thermodynamics calculations show that, if surface diffusion were the dominant factor, the last two groups would show equal strengthening. Our experimental results refute this, instead demonstrating a 100% increase in mean yield strength with increased particle size and no statistically significant increase in strength due to the addition of a coating. A separate analysis of stable plastic flow corroborates the findings, showing an order-of-magnitude increase in the rate of dislocation nucleation with a change in particle size and no change with coating. Taken together, these results demonstrate that surface diffusion plays a far smaller role in the failure of nanoparticles by dislocations as compared to geometric factors that reduce the energy barrier for dislocation nucleation.

## Introduction

Metal nanoparticles have unique properties that differ from bulk samples of the same material; for example, metal nanoparticles have high surface activity, unique optical properties, and few structural defects due to their small size. These properties are leveraged in industrial applications ranging from catalysis,^1,2^ biomedical sensors,^3,4^ and solar energy^5^ to lubricant additives^6−8^ and micro- and nanoelectromechanical devices.^9,10^ However, these applications commonly require very small nanoparticles, with diameters of 20 nm or less.^11^ At this scale, the large surface-to-volume ratio, high-curvature surfaces, and the high diffusivity of surface atoms can soften the particles and render them mechanically unstable.^12^ Therefore, a fundamental understanding of the mechanical behavior of small metal nanoparticles is needed to control their strength and stability over time.

Prior investigations have demonstrated size-dependent mechanical behavior of micro- and nanostructures with varied geometries, including particles,^13−15^ pillars,^16,17^ and wires.^18−20^ In the past, *in situ* electron microscopy was used to perform mechanical tests of these structures across a range of sizes. A key finding from these tests is the smaller-is-stronger trend, where the strength (e.g., yield strength and/or critical resolved shear stress) increases as the characteristic dimension of the structures decreases from approximately 1 μm down to approximately 50 nm.^21−26^ This behavior also occurs in polycrystalline materials and even in nanopillars with pre-existing dislocations (typically generated by sample creation using a focused ion beam),^26^ where yield strength shows an inverse dependence on size following the classical Hall–Petch relationship.^27,28^ However, conventional explanations of the dislocation pileup at grain boundaries do not apply to nanoparticles and nanopillars. Instead, Greer et al. proposed the concept of dislocation starvation,^29−31^ where the number of dislocations and sources decreases as the structures shrink to nanoscopic sizes. The overall result is that small-sized structures are nearly defect-free, such that few pre-existing dislocations or sources exist to facilitate plastic deformation. This dislocation starvation leads to ultrahigh strength, often above 1 GPa.^25^

Eventually, as the size of the particle drops below approximately 50 nm, there is a change in the size-dependent trends and the underlying physics that govern the deformation. This is because the surface provides energetically favorable sites for the heterogeneous nucleation of dislocations. It is well established that the surface nucleation of dislocations is a stress-assisted thermally activated process,^32,33^ which has been extensively studied, e.g., as a function of particle size,^34,35^ crystal orientation,^35^ internal defects like twin structure,^36−38^ surface stress,^39^ grain boundaries (for polycrystalline materials),^40,41^ and surface termination.^42^ The effect of particle size, in particular, has several geometric contributions to the strength, which can be difficult to disentangle. First, smaller structures have less surface area to provide nucleation sites.^32,43,44^ Second, prior simulations^15,34,45,46^ and experiments^15^ suggest that the angle of corners between adjacent facets affects dislocation nucleation; sharper angles (which usually exist in small nanoparticles due to more curved surfaces) lead to smaller line lengths of the embryonic dislocation and thus significantly reduced barriers to nucleation.^34,47^

However, it has also been shown that surface diffusion plays a role in dislocation nucleation. For example, experimental investigations of metal nanowires with diameters less than 100 nm^34,42^ showed that the measured energy barrier for dislocation nucleation was far lower than that predicted by simulations of dislocation nucleation in face-centered-cubic (FCC) materials^32^ but was comparable to the barrier for self-diffusion on the surface.^48^ A study of silver and platinum nanowires^49^ also suggested cooperative behavior between surface diffusion and defect nucleation. Finally, a separate study on nanowires showed a smaller-is-stronger trend for platinum that continued all the way down to 4 nm, whereas silver switched to a smaller-is-weaker trend at 15 nm.^12^ The authors attributed this different behavior to the significant difference in melting temperature, where room temperature represents a much higher homologous temperature for lower-melting-point silver, compared to platinum. Furthermore, unlike the separate work that showed Coble-creep-like deformation in ultrasmall nanoparticles^50^ and in nanotips,^51^ this investigation showed that silver’s reduction in strength happened even in the regime where deformation was controlled by dislocation nucleation. For these reasons, the authors concluded that “diffusion-assisted displacive deformation” plays a dominant role in size-dependent weakening. The explanation was that the random thermal vibration of atoms would cause kink sites to form, thus directly nucleating surface dislocations. As the particle size decreases for a specific material, the melting temperature decreases and thermal vibration increases; therefore, surface diffusion will play an increasingly strong role in nucleating dislocations. In addition, prior work by some of the present authors demonstrated both “displacive” and “diffusive” behavior of platinum nanoparticles, with a strong decrease in strength due to decreasing size for the surface-dislocation-nucleation regime (displacive deformation);^52^ however, that work, and the others mentioned, could not distinguish the mechanism of the weakening.

In summary, there are two different effects of particle size on displacive deformation: (1) geometric effects, such as higher curvature and more step edges, which cause a reduced activation barrier for dislocation nucleation, and (2) surface diffusion, where increased atom mobility leads to a higher rate of nucleating dislocations through random thermal vibration. These two effects are difficult to disentangle and, despite extensive investigations into the deformation and strength of nanostructures, the critical factors governing size-dependent surface-dislocation nucleation are still not clear. The purpose of this investigation was to distinguish between the geometric effects and the surface-diffusion effects for platinum nanoparticles. We synthesized nanoparticles in three different groups: 12 nm particles, 16 nm particles, and 12 nm particles coated with silicon oxide. This enables the separate evaluation of a change in particle size (12 nm diameter compared with 16 nm diameter), which affects both geometry and surface diffusion, and a change in surface coating (12 nm bare particles and 12 nm silica-coated particles), which primarily affects surface diffusion and not particle geometry. We then performed *in situ* compression testing in a transmission electron microscope (TEM) on all nanoparticles in these different groups to measure their strength and deformation behavior. The goal was to determine which factor—particle size or surface coating—had a more significant effect and thus which factor—geometric contributions or diffusive contributions—dominated nanoparticle deformation.

## Results and Discussion

### Direct Observation of Nanoparticle Deformation with Varying Size and Surface Coating

**Figure 1:**
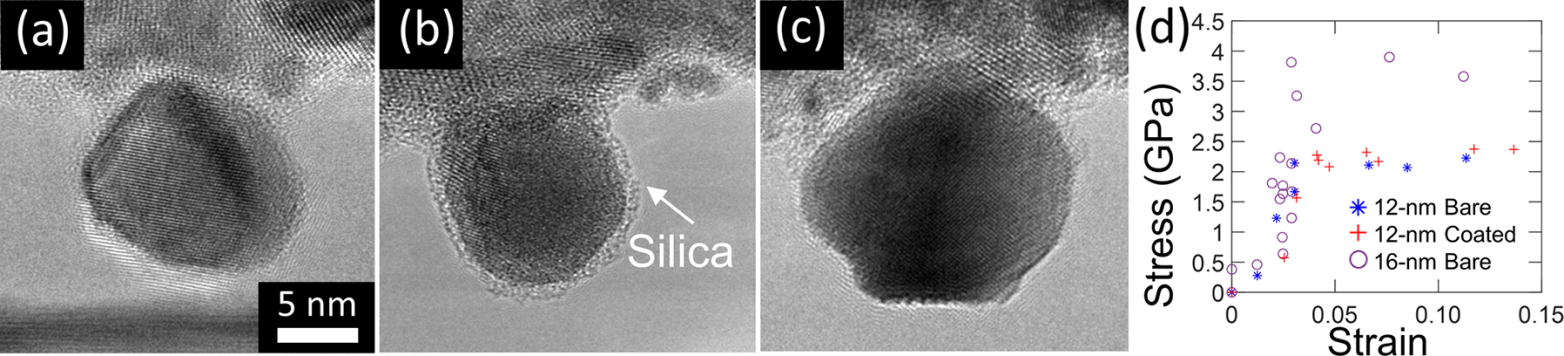
*In situ* compression was performed on three categories of particles: smaller bare nanoparticles, smaller coated nanoparticles, and larger bare nanoparticles. (a) “Smaller” bare nanoparticles have a diameter of approximately 12 nm. (b) “Coated” refers to a silica overlayer deposited after synthesis. (c) “Larger” nanoparticles have a diameter of approximately 16 nm. The detailed deformation process of these three nanoparticles is shown in Section S2.1 in the Supporting Information. (d) In all cases, the true stress–strain curves were determined.

*In situ* compression testing was performed on 39 platinum nanoparticles to investigate their mechanical behavior. Nanoparticles were deposited on a wedge-like substrate so that they could be independently accessed by an atomic force microscopy (AFM) probe that was used as an indenter. The detailed synthesis and characterization methodology is presented in the Experimental and Analysis Methods as well as Section S1 in the Supporting Information. There was natural variability in the size and shape of the synthesized nanoparticles,^53^ but the study design was to select particles for testing that fell into three categories: 12 nm bare nanoparticles (average size of 12.2 ± 1.7 nm; 14 samples), 12 nm coated nanoparticles (11.8 ± 1.7 nm; 13 samples), and 16 nm bare nanoparticles (15.7 ± 1.1 nm; 12 samples). These three groups were selected to isolate the effects of the particle size and surface termination. Images of representative particles from each group are shown in Figure 1a–c. Figure 1d shows measured stress–strain curves of these representative nanoparticles; the curves suggest that they all undergo elastic deformation up to strains of more than 3% before reaching a critical stress at which yielding occurs, as expected for dislocation-mediated deformation.^21−25^ However, the larger nanoparticles typically exhibited greater yield strengths than the smaller ones, while bare and coated nanoparticles of similar size showed little difference in mechanical response. The measured yield strengths of all of the tested nanoparticles are summarized in Section S2.1 in the Supporting Information.

**Figure 2:**
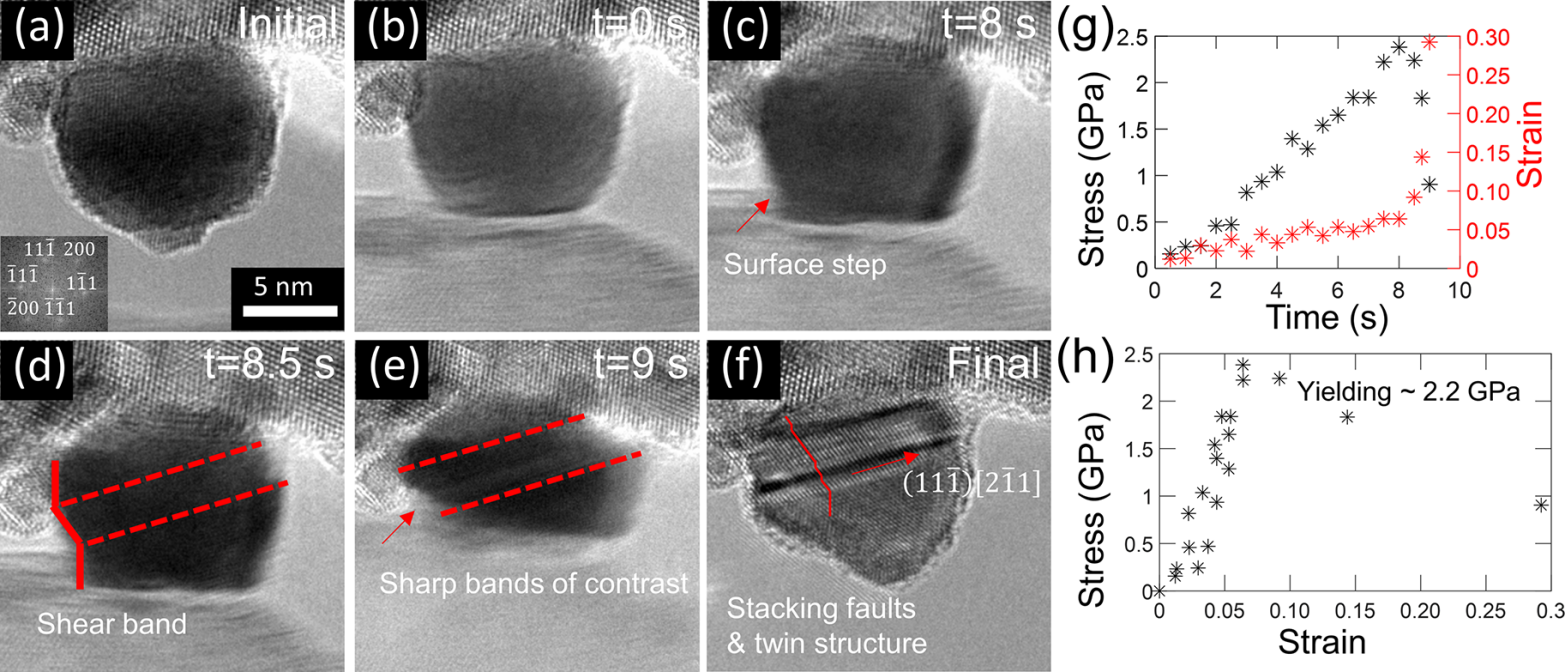
Nanoparticles failed primarily by displacive deformation. One illustrative particle is shown here (a) in its initial state, (b–e) at various stages of testing, and (f) after testing. The crystal orientation is indicated in the Fourier transform image (inset in (a)). (g, h) The true stress and strain were measured from video frames. This particle shows evidence of localized displacement, including displacement kinks in the side surfaces (red solid line in (d)), sharp bands of diffraction contrast (dashed red lines), and evidence of deformation twins after unloading (the solid line in (f) indicates changes in orientation of the (111) planes across twin structures). The majority of particles, regardless of size (12 or 16 nm) or surface condition (bare or silica-coated), showed one or more of these signatures of displacive deformation. Video S1 shows the compression of this nanoparticle in full.

The high-resolution images from the *in situ* tests confirmed that the mechanical behavior of the tested nanoparticles was dominated by defect plasticity. A frame-by-frame analysis of one 12 nm bare particle is shown in Figure 2 to provide examples of the criteria used to determine that the failure mechanism was defect plasticity. At the initial stage, the nanoparticle maintained its shape with little deformation (Figure 2a–b). The high-resolution video revealed several features indicative of dislocation-mediated deformation after yielding, as shown in Figure 2c–f. A surface step appeared, which is typical of dislocation nucleation. Furthermore, a shear band formed, and the nanoparticle underwent rapid shearing with sharp bands of contrast clearly appearing during plastic deformation. This behavior was similar to prior investigations^36,54−56^ showing surface-dislocation-nucleation events in nanowires. The stress and strain (Figure 2g) had an approximately linear relationship, characteristic of elastic deformation, which persisted until a gross shape change occurred. This particle was chosen as an illustrative example because it clearly exhibited all of the typical features of defect plasticity; the majority of particles showed one or more of these features. No differences were observed in deformation mechanism among all particles, regardless of size or surface coating.

Image analysis after detachment in Figure 2f indicated that the slip direction was consistent with (111)[211]. Combined with features like stacking faults and twin structure, this slip direction proved the existence of Shockley partial dislocations during yielding. This can be compared to prior investigations into nanoparticle deformation. While for nanograined bulk platinum with grain boundaries, 10 nm is the transition size from full dislocation to partial dislocation,^57^ individual nanostructures with free surfaces (not grain boundaries) typically show partial dislocation activity rather than full dislocation activity at sizes below 100 nm in experimental and simulation investigations.^12,37,38,58−60^ Besides surface dislocation nucleation, a previous study on platinum nanowires by Wang et al. suggested that a nanowire may exhibit full dislocations (nucleated in the bulk) along with partial dislocations (nucleated at the surface), and these may interact inside the material.^61^ Similar behavior was not observed in these particles, likely because the small size of the nanoparticles (<20 nm in all directions) means that there is far less “bulk” material as compared to a long nanowire.

The yielding occurred for this particle at 2.2 GPa (Figure 2h), an ultrahigh stress that has been observed in defect-free single crystals. The diffraction fringes in the initial and final high-resolution images also allow the analysis of critical resolved shear stress τ_c_ = σ_*y*_ cos Φ cos λ, where σ_*y*_ is the yield stress, cos Φ cos λ is the Schmid factor, Φ is the angle between the force direction and shear plane’s normal vector [111], and λ is the angle between the force direction and the shear direction [211], based on Schmid’s law.^62^ The force direction measured during yielding is [311]. Therefore, the critical resolved shear stress is 0.94 GPa. This result is also consistent with a previous measurement of an 11.5 nm platinum nanoparticle.^63^ While the yield strength measurement is loading-direction-dependent, a simple calculation of all possible loading directions (Section S2.2 in the Supporting Information) shows that the maximum Schmid factor varies little, with an average value of 0.462 and a standard deviation of 0.033. Therefore, the orientation of the particle is expected to have only a minor effect on measured yield strength. For this reason, and to be consistent with other similar investigations, the measured yield strength is reported instead of the resolved shear strength for the remainder of the discussion.

### Interrogating the Effect of Surface Coating and Particle Size on Yield Strength

**Figure 3:**
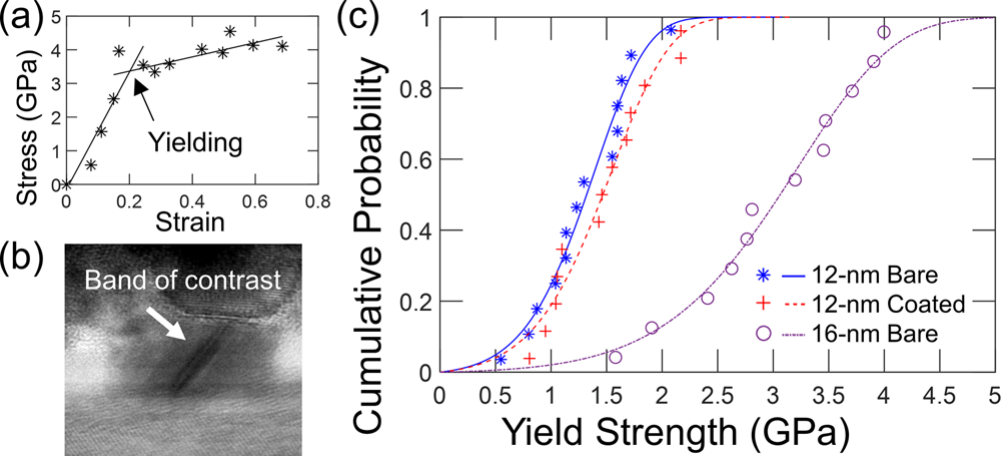
Measurements of yield strength demonstrate little effect of coating, but a large effect of particle size. The yield strength is determined from either (a) a kink in the stress–strain curve or (b) the appearance in the video frames of bands of contrast which are characteristic features of dislocation activity. (c) The yield strengths, corrected for stress concentration (see main text), are shown for all tested nanoparticles in the form of cumulative distribution functions, where the symbols are measured data and the corresponding lines are fits to eq 7, from transition state theory.

The yield strengths of all tested nanoparticles were determined to understand the effect of the size and coating on the surface nucleation of dislocations. Either of the following two criteria was used to identify the yield point. First, using the stress–strain curve, data points in the elastic deformation stage were fit with a linear trend, as were the data points in the plastic-deformation stage; the intersection of these two linear fits was identified as the moment when yielding happens, as shown in Figure 3a. Second, if no clear transition was observed between the elastic deformation and plastic deformation stages, then video analysis was used to find the earliest frames with characteristic features of yielding, such as those shown in Figure 2d–f or Figure 3b, suggesting dislocation-associated slip. Mordehai et al. showed that the stress distribution in a nanoparticle is nonuniform and can have stress concentration at the edges of contact.^13^ We used this model to correct the measured yield strength in order to account for this effect (Section S3.1, Supporting Information). The results for raw and corrected yield strengths are compared and discussed in Section S3.1; the absolute values of measured parameters differ, but the significant differences between groups persist in both the raw and corrected values.

A range of yield strengths was observed for each population of particles. The mean yield strength of the 12 nm bare particles was 1.30 ± 0.42 GPa. The 16 nm bare particles were significantly stronger with a yield strength of 2.99 ± 0.77 GPa. By contrast, there was no statistically significant difference in strength for 12 nm particles with the coating layer; the mean yield strength for these particles was 1.46 ± 0.45 GPa. The statistics of the measured yield strengths are presented in Figure 3c as a cumulative distribution function *F*(σ,*T*) (CDF), where *T* = 300 K since the particles were tested at room temperature.

To extract the relevant material parameters, surface dislocation nucleation is modeled as a thermally activated process, in accordance with prior work.^32,64^ Specifically, the nucleation rate *v* takes an Arrhenius form^65^
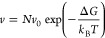
1where *N* is the number of surface dislocation nucleation sites, *v*_0_ is the attempt frequency, Δ*G* is the activation energy, *k*_B_ is Boltzmann’s constant, and *T* is the temperature. The activation energy Δ*G* decreases with increased applied stress σ and temperature *T* by^34,64^
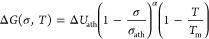
2where Δ*U*_ath_ is the zero-stress zero-temperature activation energy also called athermal activation energy, *T*_m_ is the melting temperature, and σ_ath_ is the athermal strength. While the temperature has a linear effect because of the contribution of activation entropy, the stress effect is still being investigated. The simplest case would be assuming α = 1 corresponding to a constant activation volume Ω. Technically, Ω is defined as Ω(σ) = −∂Δ*G*/∂σ; however, it too can be temperature-dependent as Ω(σ,*T*) = Ω(σ)(1 – *T*/*T*_m_). Various simulation investigations indicate that an assumption of α = 1 oversimplifies the stress-dependent activation energy, instead suggesting α = 1.5 for molybdenum nanoparticles^64^ and α = 4 for copper^32^ or palladium^34^ nanowires. In the case of α ≠ 1 where the activation volume is no longer constant, then a characteristic activation volume is defined at the most probable strength σ̅, where *∂F*(σ)/*∂*σ is maximum or ∂^2^*F*(σ)/*∂*σ^2^ = 0. To extract this quantity, we follow the approach of Chachamovitz et al. and we obtain the characteristic activation volume directly from the degree of scattering of the distribution of yield strengths^64^
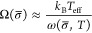
3a
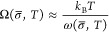
3bwhere *T*_eff_ is the effective temperature defined as 1/*T*_eff_ = 1/*T* – 1/*T*_m_ by considering the activation entropy’s contribution and ω(σ̅,*T*) is the distribution width characterizing the degree of scattering of *F*(σ) measured at the experimental temperature *T* (in this case, room temperature). The detailed calculations of σ̅, ω(σ̅,*T*), and Ω(σ̅) are described in Section S3.2 of the Supporting Information.

The exact form of *F*(σ) can be derived from the CDF of the material’s first yielding event *F*(*t*) at time *t* and at a given testing condition. *F*(*t*) is linked with its first derivative *Ḟ*(*t*), according to the model established by Mason et al.^66^
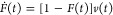
4where the probability density is necessarily proportional to the remaining unyielded population [1 – *F*(*t*)] and the time-dependent dislocation nucleation rate *v*(*t*). This leads to
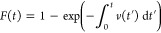
5Time and applied stress are related because in elastic deformation it is assumed that , in which *E* is the elastic modulus, and  refers to elastic strain rate.^12,32,34,42^ Hence, combined with eq 1, *F*(*t*) can be converted to the form of *F*(σ):
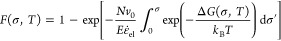
6If Δ*G* is replaced with the form of eq 2, *F*(σ) can be reduced based on the derivations from Chen et al.^34^ and Chachamovitz et al.:^64^
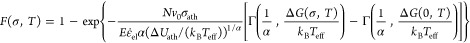
7where Γ is the incomplete upper gamma function defined as Γ(*a*,*x*)=∫_*x*_^∞^*t*^*a*^^–1^*e*^–*t*^ d*t*. By fitting this equation, the values of associated activation quantities can be extracted from the experimentally measured yield strength in the form of a CDF as shown in Figure 3c. α = 4 was selected, and the detailed description of how to extract activation parameters is documented in Section S3.3 in the Supporting Information.

The determined values of all of the activation parameters are shown in Table 1. While this numerical fitting to the CDF represents the state-of-the-art method for extracting physical quantities, this is a multiparameter fit to an exponential distribution and therefore some parameters have large uncertainty; for this reason, we’ve chosen to report them as a range of values. To evaluate the overall consistency with prior results, we first examine all 39 measurements as a whole. The characteristic activation volumes ranged over 0.2–0.5 *b*^3^, where *b* refers to the Burgers vector of the full dislocation in the FCC platinum crystal. These activation volumes are consistent with those of other nanomaterials in prior experiments.^12,34,42,67^ The estimated athermal strengths are consistent with the order of magnitude of the ideal theoretical shear strength of platinum, which is approximately 10 GPa, corresponding to *G*/2π, where *G* is the shear modulus.^68^ The estimated activation energy values are in the range of 0.2–0.3 eV, consistent with values obtained in previous experiments for other nanomaterials.^12,34,42^ While this range is still lower than the suggested range from simulation studies (>0.4 eV),^32,44,46,64^ Chen et al.^34^ have demonstrated that pre-existing flaws in nanostructures reduce the activation energy. Unlike the atomically flat surfaces that are common in simulations, the surface of the nanoparticles is directly observed to have steps, kinks, and irregular geometry. The estimated prefactor values *Nv*_0_ are in the orders of magnitude of 10–1000 s^–1^. This is far below the values determined in simulation studies with attempt frequency on the order of the Debye frequency (∼10^13^ s^–1^),^32,44,64^ but the measured values are close to experimentally measurements for other nanostructures.^12,34,42^ This has been attributed to the fact that surface dislocation nucleation involves the collective action of a group of atoms, which significantly lowers the attempt frequency compared to a single-atom process.

**Table 1:** Activation Parameters Extracted by Fitting the Cumulative Distribution Function of Measured Yield Strength^a^

	most probable strength σ̅ (GPa)	activation volume Ω(σ̅) (*b*)^3^	athermal strength σ_ath_ (GPa)	athermal activation energy Δ*U*_ath_ (eV)	prefactor *Nv*_*0*_ (s^–1^)
12 nm bare	1.30 ± 0.01	0.487 ± 0.023	6.34–11.02	0.218–0.277	34.9–435.4
12 nm coated	1.46 ± 0.01	0.435 ± 0.009	6.96–11.81	0.216–0.270	40.7–430.2
16 nm bare	3.03 ± 0.01	0.262 ± 0.006	15.21–23.02	0.275–0.327	68.0–678.0

a The most probable strength and the characteristic activation volume are computed directly from the distribution of yield strengths and are quoted as best-fit values with uncertainty. All other parameters require multiple-parameter numerical fitting to the cumulative distribution function, as described in the main text, and are quoted as a range of possible values.

The key result of this analysis is a comparison of the activation parameters across the three different groups. The measured value for most-probable strength is significantly higher for large nanoparticles but indistinguishable between coated and bare nanoparticles at the smaller sizes. The activation volume, which has the physical meaning of sensitivity to stress, is reduced by almost 50% due to an increase in size, whereas the addition of a surface coating reduced it by less than 11%. Additionally, the athermal strengths were virtually identical for bare and coated nanoparticles at 12 nm size yet significantly different for the 16 nm particles. Similarly, the ranges of athermal activation energy showed no change with coating yet a meaningful change with particle size. While the prefactor was also observed to increase for the 16 nm particles as compared to the others, the range of uncertainty for this parameter is too large to merit meaningful conclusions. Taken together, this comparison of activation parameters for the three groups demonstrates a meaningful change in the physics of dislocation nucleation with a change in particle size and no change with the addition of a surface coating.

### Understanding the Rate of Dislocation Nucleation across the Groups of Particles

**Figure 4:**
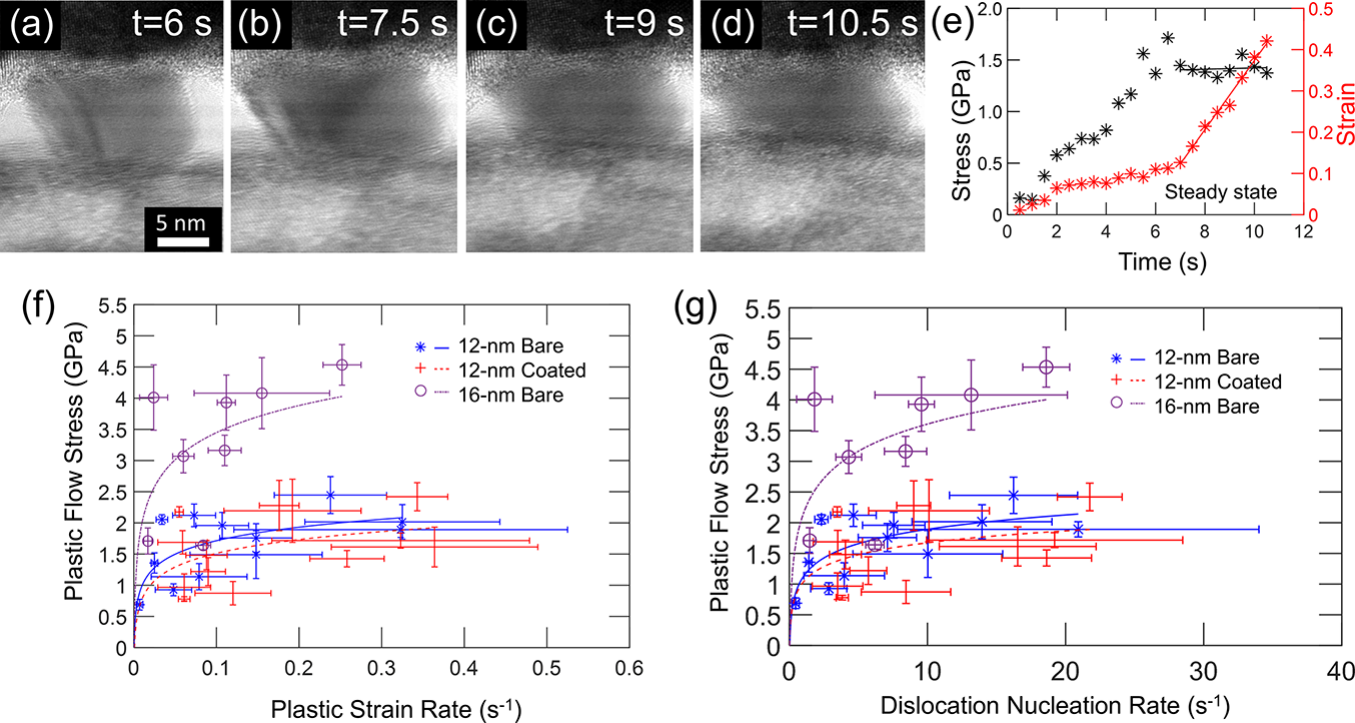
Measurements of stress during plastic flow demonstrate the relationship between plastic strain rate and flow stress. (a–d) After yielding, most nanoparticles deform continuously. (e) Their measured strain increases approximately linearly (red line, whose slope determines the plastic strain rate) at a near-constant stress (black line, whose magnitude determines the plastic flow stress). (f) The plastic flow stress and plastic strain rate are plotted with curves predicted by the Arrhenius equation (eq 8). (g) The dislocation nucleation rate can also be computed (eq 9a) from the plastic flow stress. All uncertainties are given by a 95% confidence interval.

After yielding, many nanoparticles deformed plastically in a continuous manner, as shown in Figure 4a–d, exhibiting significant plastic flow. The plots of stress and strain as functions of time (Figure 4e) suggest continuous deformation, where the nanoparticle reaches something like a steady-state condition at a constant strain rate and the stress is approximately constant. In general, the AFM probe’s cantilevers are softer than the particles, and so the compression occurs under approximately load-control conditions. The plastic flow stress is defined as the average stress in the postelastic region where the strain rate is approximately linear; it is similar to, but not necessarily equal to, the yield strength. Analogous to yield-strength measurements, the plastic flow stress is also corrected to account for the nonuniform stress distribution, as described in Section S3.1 of the Supporting Information. For all nanoparticles that did not fail catastrophically (i.e., a rapid increase in strain and corresponding drop in stress, similar to Figure 2(g−h) shows) but rather showed some continuous plastic flow, the steady-state flow stress and corresponding plastic strain rate are plotted in Figure 4f. Taken as a group, the mean plastic flow stress of the bare and coated nanoparticles of 12 nm diameters are indistinguishable, at 1.65 ± 0.53 and 1.60 ± 0.55 GPa, respectively. By contrast, the mean plastic flow stress of the 16 nm bare particle was 3.27 ± 1.09 GPa, twice as large as that of the smaller bare particle.

The trend in the plot of flow stress versus plastic strain rate in Figure 4f can be empirically described by the Arrhenius equation^68,69^
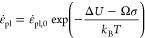
8where  is the plastic strain rate,  is a constant containing the pre-exponential factors in eq 1, and Δ*U* is the maximum activation energy at the testing condition. Note that this empirical equation is not as sophisticated as the CDF analysis of first-yielding that was presented in the prior section and makes the assumption of a constant activation volume. However, this analysis represents an additional independent method for analyzing the activation parameters of nanoparticle deformation. The rate of stable plastic flow at a given stress can be related to the rate of dislocation nucleation *v*, by estimating the amount of plastic deformation per unit time contributed by each dislocation nucleation event using
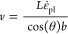
9a
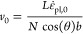
9bwhere *L* is the initial height of the particle, θ is the angle between the slip direction and the loading direction (approximated as 45°), and *b* is the Burgers vector of the full dislocation in the platinum crystal. The calculated dislocation nucleation rate of the present testing ranges from 0.4 to 22 s^–1^ as shown in Figure 4g.

The Arrhenius equation can be fit to the data based on Figure 4f (or based on Figure 4g). This fitting yields an activation volume of 0.584*b*^3^ (or 0.542*b*^3^, for fitting to Figure 4g) for the 12 nm bare particles. An increase in size to 16 nm particles causes a significant change in activation volume to 0.292*b*^3^ (0.286*b*^3^). By contrast, the addition of a surface coating to the 12 nm particle yields a much smaller change, to 0.576*b*^3^ (0.653*b*^3^). The activation energy Δ*U* can also be estimated from the intercept of the logarithmic plot in Figure 4g, if the prefactor *Nv*_0_ is set to the calculated values in Table 1. This leads to the estimated activation energy in the range of 0.178–0.243, 0.191–0.252, and 0.196–0.255 eV for 12 nm bare, 12 nm coated, and 16 nm bare nanoparticles, respectively. The measured values differ from those of Table 1, which were extracted from a fit to the cumulative distribution function of the first-yield event. This difference might be attributed to the fact that subsequent nucleation events differ from the initial one, as the first-yield event can modify the surface morphology and make it easier for further dislocation nucleation.^34^ Regardless, the *trends* in behavior across different groups are consistent between this analysis and those of the prior section; namely, that the effect of increasing the size of the nanoparticle dwarfed the effect of adding a surface coating.

### Distinguishing the Effect of Surface Mobility from That of Geometric Factors in the Surface Nucleation of Dislocations

As described previously, there are two different factors governing the surface nucleation of dislocations in small nanostructures: geometric factors, where smaller particles have larger curvature and therefore a reduced activation barrier for a dislocation to nucleate, and surface diffusion, where small particles have lower effective melting temperatures and higher surface mobility, by which the random thermal motion causes kinks to form, nucleating dislocations at the reduced activation barrier. It should be noted that surface diffusion,^50,52,70^ or grain-boundary sliding which occurs between two adjacent grains in a polycrystalline material,^71^ has also been suggested to cause gross atom migration, resulting in significant material deformation without dislocations. For nanostructures, this is often called “diffusive deformation”. Prior work by the present authors using a similar apparatus^52^ have explicitly distinguished homogeneous deformation in the “diffusive regime” from heterogeneous deformation in the “displacive regime”, with a transition occurring at approximately 9 nm. Therefore, that prior work, and also prior simulations of the so-called “Coble-creep-like behavior”,^51^ confirm that the present 12 and 16 nm nanoparticles should undergo displacive (defect-based) deformation. Therefore, the present study aims specifically to evaluate the postulated effect of surface diffusion on the surface nucleation of dislocations.

To understand the mechanisms governing failure of nanoparticles, it is useful to distinguish between the entropic contribution and the geometric contribution to the surface nucleation of dislocations. The entropic effect is described by the temperature part of the activation energy in eq 2, namely Δ*G*(0,*T*) = Δ*U*_ath_(1 – *T*/*T*_m_). The geometric effects are described by changes to the characteristic activation volume at a given temperature or to the athermal activation energy (or athermal activation strength), which do not depend on homologous temperature.

To compute the thermal effects, we need to account for the fact that, although all of the experimental tests were performed in room-temperature conditions, the homologous temperatures of nanoparticles from the three groups are not the same. In other words, the entropies Δ*U*_ath_/*T*_m_ or the degrees of surface disorder are different for nanoparticles in these three groups because their melting temperature varies. The coating passivates the surface and improves the thermal stability; therefore, the melting temperature of coated nanoparticles should be greater than that of bare nanoparticles. From an atomistic perspective, atoms on a free surface have high activity as they are not fully bonded by surrounding atoms, unlike atoms in the bulk volume. For the 12 nm coated particles, the surface atoms of the nanoparticle are bonded not only with atoms in the volume but also atoms or molecules belonging to the coating. For the 16 nm bare particles, the larger size also leads to an increase in thermal stability because the fraction of atoms belonging to the surface decreases as size increases. The effect of size and coating on melting temperature of nanoparticles has been quantitatively analyzed based on the model established by Shi,^72^ later developed by Jiang et al.,^82,83^ and further applied by Liang et al.^73^ in analysis of surface termination:
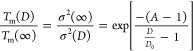
10where *T*_m_(*D*) is the melting temperature of a nanoparticle at characteristic size *D*, *T*_m_(∞) is the melting temperature of the corresponding bulk material, σ^2^(*D*) and σ^2^(∞) are the averaged mean–square displacements (MSDs) of all atoms in the nanoparticle at characteristic size *D* and the corresponding bulk material, respectively, the parameter *A* is a constant ratio of the MSD of the surface atoms of the nanoparticle to that of the atoms in the volume of the nanoparticle, and *D*_0_ is the critical size at which almost all the atoms are on the surface.

For bare and coated nanoparticles of the same size, the difference in melting temperature is primarily governed by *A* because the coating passivates the surface and reduces the mean-square displacement of surface atoms. Once the fraction of surface atoms that are effectively passivated by the coating is determined, the increase in melting temperature of the nanoparticle due to the surface coating can be estimated (Supporting Information, Section S4). The conservatively estimated values of *A* for bare platinum nanoparticles and silica-coated platinum nanoparticles are about 1.83 and 1.59, respectively. This corresponds to approximately 30% of the surface atoms being effectively passivated by the silica layer. Using eq 10, the predicted melting temperature of the 12 nm bare nanoparticles is 1793 K; the predicted melting temperature of the 12 nm coated nanoparticles is 1875 K, while that of 16 nm bare nanoparticles is 1862 K (Section S4, Supporting Information). According to Δ*G*(0,*T*) = Δ*U*_ath_(1 – *T*/*T*_m_) with (*T* = 300 K), the 12 nm bare, 12 nm coated, and 16 nm bare nanoparticles have 83%, 84%, and 84% of the athermal activation energy, respectively. This change is very small, but this analysis indicates a nearly identical change in strength for coated and larger nanoparticles. This calculation leads to a firm and testable result: based on thermal effects alone, the effect of adding a coating to the 12 nm nanoparticle and of increasing particle size from 12 to 16 nm should lead to similar increases in experimentally measured strengths. If thermal effects, such as surface diffusion, play a dominant role in the surface nucleation of dislocations, then the difference in strength and nucleation rate would be expected to be similar for coated and large-size nanoparticles. Instead, the increase in strength that corresponds to the larger size dwarfs the increase due to the coating. The same conclusion was reached by both of the quantitative analyses—from the CDF analysis of yield strength and from the dislocation nucleation rate of continuous plastic flow.

Within the geometric mechanisms, the effect of surface curvature on barrier height can be compared with the effect of a reduced number of activation sites for smaller particles. As discussed in the Introduction, smaller nanostructures also have higher curvature and more surface steps that are preferred sites for dislocation nucleation,^59^ reducing activation energy^74^ and strength,^75,76^ but they also have fewer available nucleation sites for surface dislocation, since the total surface area decreases as the size decreases.^32,43,44^ The former contribution has an exponential effect on strength, while the latter contribution is linear, according to eq 1. Our experimental results show a 100% increase in mean strength of the 16 nm particle and significant increases in athermal activation energy and athermal strength as compared to 12 nm particles. This difference cannot be explained by the estimated 78% increase in surface area (square of nanoparticle size) associated with this change in size. Furthermore, there is no appreciable change in prefactor *Nv*_0_, within the uncertainty of the measurement, as would be expected if the number of nucleation sites played an important role. Therefore, the geometric contributions to barrier reduction associated with the curvature of small particles is demonstrated to have the strongest effect on surface dislocation nucleation.

## Conclusions

In summary, we explored the role of size and surface termination in the mechanical behavior of platinum nanoparticles by *in situ* TEM. A reference group of bare 12 nm nanoparticles were compared against two test groups: bare 16 nm nanoparticles and 12 nm nanoparticles with a surface coating. An analysis of surface-atom mobility showed that the change in size and surface termination should have had a small but nearly identical effect on the melting temperatures of the two test groups. Therefore, if thermal effects and surface diffusion played a dominant role in defect nucleation and yield strength, then these test groups would show a similar increase in strength. By contrast, the large nanoparticles were twice as strong as smaller nanoparticles, while the coating resulted in no statistically significant difference in yield strength. Likewise, the calculated rate of dislocation nucleation was reduced by an order of magnitude at the same plastic flow stress in the larger particles, while there was virtually no change with the addition of a surface coating. Taken together, these results suggest that geometric effects of surface curvature play a far stronger role in the surface nucleation of dislocations, compared to entropic effects and surface diffusion. The results help to disentangle these two contributions and advance the understanding of the failure of metal nanostructures.

## Experimental and Analysis Methods

### Sample Synthesis

Wedge-like silicon substrates (<200 nm plateau wedge, Bruker, Billerica, MA) were plasma-cleaned and coated with a 20 nm cerium oxide layer through sputtering (Nexdep, Angstrom Engineering, Kitchener, Canada), because cerium oxide is more thermodynamically stable than silicon oxide. Then, a thin layer of platinum was deposited on the top surface of the substrate through electron-beam evaporation (MEB 550-S, Plassys, Marolles-en-Hurepoix, France). Afterward, the sample was annealed in an air atmosphere in a furnace (Thermolyne 1200 °C 7 × 5 × 10 in., ThermoFisher Scientific, Waltham, MA), and the continuous thin layer underwent dewetting to form nanoparticles.^77,78^ Several combinations of deposition thickness and annealing temperature were tested to determine the optimal synthesis recipe (Section S1.1, Supporting Information). Nanoparticles in this research were synthesized with 0.5 nm deposition thickness and an annealing temperature of 630 °C, to obtain nanoparticles in the desired size range of 12–16 nm. For the coated sample, there was one extra step after nanoparticle synthesis, namely adding a thin (1−3 nm) layer of silicon oxide through sputtering.

### *In Situ* Testing and Analysis

The *in situ* compression testing was performed inside a mechanical testing holder (Biasing Manipulator Model 1800, Hummingbird Scientific, Lacey, WA) inside of a TEM (Titan Themis G2 200, Thermo Fisher Scientific, Waltham, MA) at an accelerating voltage of 200 kV. A commercial AFM probe (Tap300DLC, BudgetSensors, Sofia, Bulgaria), with a nominal stiffness constant of 40 N/m, was used as the indenter. For accurate force measurement, the AFM probes were calibrated, and the stiffness constant was computed following Sader’s method^79−81^ (Section S1.2, Supporting Information). The prepared wedge-like substrate and the calibrated AFM probe were mounted on opposite sides of the holder. The loading speed was controlled by the motion of the piezo tube and, in this work, ranged from 0.5 to 5 nm/s. The real-time *in situ* experiments were recorded by a charge-coupled-device camera at a rate of 0.25 s/frame. The postprocessing of the video was carried out using ImageJ. The deformation and strain of nanoparticles during compression were measured in the individual video frames. The force was calculated from the deflection of the AFM probe. Section S1.3 of the Supporting Information elaborates on how the *in situ* videos were analyzed. In addition, the environmental temperature was not appreciably raised above room temperature because the beam current was maintained at a level below 30 A/cm^2^ such that the beam had no observable effect on bare nanoparticles (Section S1.4, Supporting Information). Because silica is known to undergo electron-beam-induced material deformation, the coated nanoparticles were exposed to the electron beam for a sufficient time to eliminate any transient behavior (as described in Section S1.5, Supporting Information).

## Data Availability

All data associated with this manuscript is publicly available through the University of Pittsburgh’s online repository, D–Scholarship. It is accessible via DOI by using the following link: 10.18117/qgkb-g857.

## Supporting Information Available

Experimental methods (which contains details of sample synthesis, probe calibration, compression testing, effects of the electron beam); more details regarding mechanical behavior of all nanoparticles (which contains a more comprehensive presentation of the mechanical-test data and an analysis of the effect of loading direction); detailed data analysis from cumulative distribution function (which discusses how to correct the yield strength due to nonuniform stress distributions in nanoparticles, the detailed procedure for extracting, activation volume, most-probable strength, and athermal strength and athermal energy); and melting temperature of bare and coated nanoparticles (PDF)
Mechanical behavior of the nanoparticle in Figure 2 (AVI).

## Supplementary Material

nn3c09026_si_001.pdf

nn3c09026_si_002.avi
