# Supplementary material for: Separating Geometric and Diffusive Contributions to the Surface Nucleation of Dislocations in Nanoparticles
Source: ACS Nano. 2024 Jan 26;18(5):4170–9. doi: 10.1021/acsnano.3c09026 (PMC10851666; doi:10.1021/acsnano.3c09026)
Supplement: Supplementary file 1 — nn3c09026_si_001.pdf [file nn3c09026_si_001.pdf]

Supporting Information for:

Separating geometric and diffusive contributions to the surface nucleation of dislocations in nanoparticles

Ruikang Ding,[1] Soodabeh Azadehranjbar,[1] Ingrid M. Padilla Espinosa,[2] Ashlie Martini,[2] Tevis D. B. Jacobs[1*]

[1)]Department of Mechanical Engineering and Materials Science, University of Pittsburgh, Pittsburgh, PA 15261, USA

[2)]Department of Mechanical Engineering, University of California, Merced, Merced, CA 95340, USA

S1. Experimental Methods

S1.1. Sample Synthesis

Different recipes with varied deposition thickness and post-annealing temperature were tested for the synthesis of platinum nanoparticles as shown in Figure S1. In all recipes, the platinum deposition rate was 0.03 nm/s, the average heating rate was 10 ℃/min, and the annealing time was 30 min at the temperature indicated in Figure S1. The nanoparticle size and interparticle spacing were assessed with variations in the deposition recipes, and it was found that higher annealing temperatures and thicker deposition layers tended to produce larger nanoparticles, as expected.

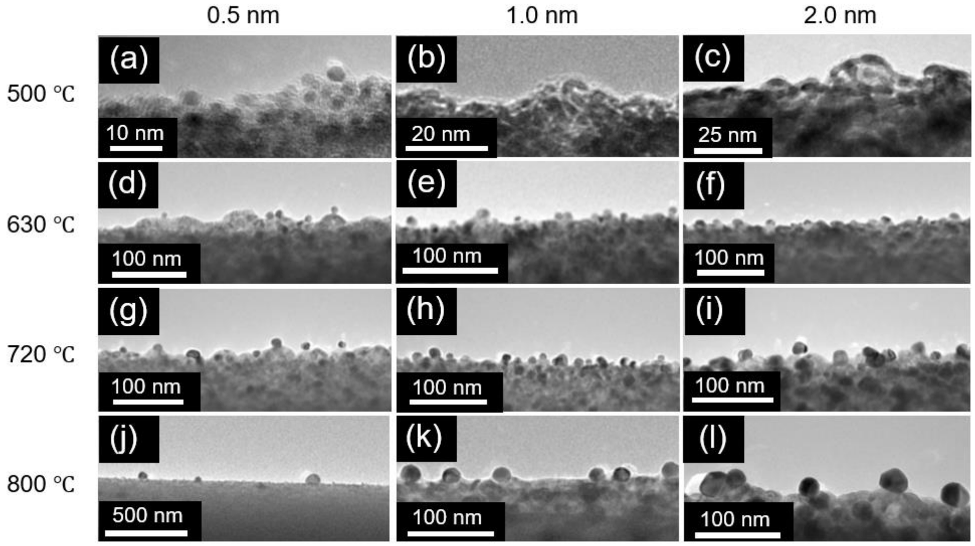

Figure S1: Different combinations of deposition thickness and post-annealing temperature produced nanoparticles with different size and interparticle spacing. (a–l) Typical side–view TEM images of the wedge–like substrate's top surface show the characteristics of nanoparticle distribution from 12 different combinations. Corresponding values of deposition thickness and annealing temperature are labeled at the top and left sides of the figure, respectively.

To assess each recipe, three low–magnification images were taken for each synthesis condition and used to get the statistics of nanoparticle distribution. The measured average values of nanoparticle size and interparticle spacing on the top surface for all 12 conditions are summarized in Table S1. To enable the *in situ* testing, the testable nanoparticles needed to be on the top surface of the wedge and spaced sufficiently far from neighboring particles (approximately at least 20 nm) such that they could be independently accessed. Eventually it was determined that the nanoparticles synthesized with a 0.5-nm deposition thickness and an annealing temperature of 630 ℃

produced optimal particles for this research, as the size distribution was within the target range and the density of testable nanoparticles was also acceptable.

Table S1: Size and interparticle spacing of platinum nanoparticles synthesized by the 12 recipes are summarized. Data are represented as mean plus and minus standard deviation.

Temperature	Thickness	Nanoparticle Size	Nanoparticle Interspacing	Testable Nanoparticle Size	Testable Nanoparticle Interspacing
500 ℃	0.5 nm	2.5±0.5 nm	4.7±0.9 nm	3.2±0.5 nm	>1 μm
	1.0 nm	3.1±0.3 nm	6.0±4.5 nm	-	-
	2.0 nm	4.9±1.2 nm	8.4±1.6 nm	-	-
630 ℃	0.5 nm	11.4±3.3 nm	80.0±53.4 nm	11.3±2.6 nm	162.4±94.0 nm
	1.0 nm	6.2±1.4 nm	17.1±8.4 nm	7.8±2.5 nm	133.4±74.3 nm
	2.0 nm	10.2±2.7 nm	16.7±6.1 nm	13.5±2.8 nm	244.7±50.6 nm
720 ℃	0.5 nm	10.7±2.3 nm	46.3±28.3 nm	11.5±1.7 nm	186.6±70.3 nm
	1.0 nm	10.9±3.0 nm	25.7±7.7 nm	13.2±4.5 nm	194.8±145.6 nm
	2.0 nm	16.1±4.5 nm	36.8±14.7 nm	18.4±6.3 nm	129.8±117.8 nm
800 ℃	0.5 nm	35.6±18.9 nm	435.6±355.2 nm	44.5±11.8 nm	737.5±204.9 nm
	1.0 nm	16.0±2.2 nm	71.0±59.3 nm	16.4±1.1 nm	122.5±83.6 nm
	2.0 nm	28.5±6.6 nm	58.8±21.2 nm	31.5±8.0 nm	167.4±103.1 nm

S1.2. AFM Probe Calibration

The stiffness constant of AFM probes is accurately determined by using Sader's method. In this method, stiffness constant k_s is:[1–3]

$$k_s = 0.1906\rho_f W^2 L_c Q_f \Gamma_i(\omega_f)\omega_f^2 \tag{S1}$$

where ρ_f is the density of fluid (1.18 kg/m^3 for air), W is the width of the cantilever, L_c is the length of the cantilever, Q_f is the quality factor, $\Gamma_i(\omega_f)$ is the imaginary component of a hydrodynamic function, and ω_f is the angular resonant frequency. The width and length of the cantilever were measured with an optical microscope. The quality factor and resonance frequency were measured using an AFM microscope (Dimension Icon, Bruker, Billerica, MA).

S1.3. In Situ *Compression Testing*

The *in situ* testing apparatus had the configuration shown in Figure S2a. During testing in Figure S2b–c, the AFM-probe indenter approached the target nanoparticle and compressed it at a constant speed controlled by a piezoelectric actuator. The loading speed, nanoparticle size, length, deformation, and contact diameter were directly measured from the video frames. The piezo motion was pre-measured when the probe was near to the particle, but not yet making contact; this

established the velocity and magnitude of motion. Then the identical test was performed with contact of the nanoparticle, so that cantilever deflection could be computed. The nanoparticle size was determined by tracing the profiles of individual nanoparticles, and the average diameter as well as its standard deviation were extracted from the tracing.[4] The length was defined as the distance from the substrate side to the probe side of the nanoparticle, and the contact diameter refers to the width of contact between the probe and the nanoparticle. The area of contact was computed assuming a circular contact. The loading force F was computed as follows:

$$F = k_s(V\Delta t - D_d) \quad \text{(S2)}$$

where k_s is the stiffness of the AFM probe, and the terms in parentheses represent the deflection of the probe. Specifically, the real–time deflection equals the loading speed V times the change of time Δt from the initial contact, minus the real–time deformation of the nanoparticle D_d. Sometimes, the nanoparticle slipped and displaced a short distance of 1–2 nm in loading direction, which contributes extra error to the force measurement. In this circumstance, the force is corrected by subtracting the slip distance from the measured deflection. The mechanical behavior of measured nanoparticles in the main text is described by stress-and-strain-vs-time plots as well as stress/strain curves. The calculated true strain was computed as the logarithm of the instantaneous height over the original height. The true stress was computed as the calculated force divided by the real–time contact area between the particle and the indenter.

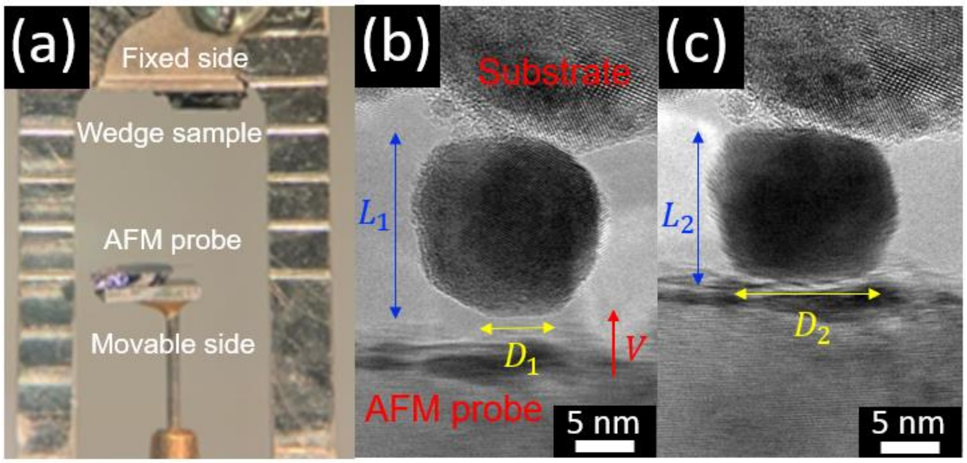

Figure S2: *In situ* testing was performed inside a TEM. (a) The apparatus had an AFM probe mounted on the movable side, which was controlled by a piezoelectric actuator, and a wedge sample with nanoparticles mounted on the fixed size. (b, c) A typical nanoparticle is shown at two different moments in the compression test. L_1 and L_2 are the length of nanoparticle, D_1 and D_2 are the contact diameter. V is the indentation speed of the AFM probe.

S1.4. The Effect of Electron Beam Intensity on Samples

The heating effect of the electron beam on the sample is analyzed in a way similar to the work of Sun *et al.*[5] based on Fisher's model:[6]

$$\Delta T = \frac{I}{4k\pi}\left(\frac{\Delta E}{d}\right)\left(1 + 2\ln\frac{r}{r_0}\right) \tag{S3}$$

where ΔT is the maximum temperature rise caused by electron beam, I is beam current, k is thermal conductivity of the sample, ΔE is the total energy loss per electron in the sample with thickness d, r is the characteristic radius of the sample, and r_0 is the beam radius. The $\frac{\Delta E}{d}$ term can be determined according to Bethe–Bloch equation:[7]

$$\frac{\Delta E}{d} = \frac{2\pi Z\rho\left(\frac{e^2}{4\pi\varepsilon_0}\right)^2}{mv_e{}^2}\left\{\ln\left[\frac{E\left(E+mc^2\right)^2\beta^2}{2I_e^2mc^2}\right] + (1-\beta^2) - \left(1-\sqrt{1-\beta^2}+\beta^2\right)\ln 2 + \frac{1}{8}\left(1-\sqrt{1-\beta^2}\right)^2\right\} \tag{S4}$$

where Z is the atomic number of the target element, ρ is the atomic density, e is charge of unit electron, ε_0 is dielectric constant, m is the electron rest mass, v_e is the electron velocity, c is the speed of light, E is electron energy, I_e is the average excitation energy of electrons (I_e=8.8×Z), and $\beta = v_e/c$. In this research, the acceleration voltage was 200 kV, which means v_e=2.084×10^8 m/s and β=0.695. r was about 1 mm, and r_0 was conservatively estimated to be 50 nm. For platinum nanoparticles, Z=78, k is 77.8 W m^{-1} K^{-1},[8] ρ is 6.6×10^{28} m^{-3} (mass density: 21.45 g/cm^3),[9] and I_e=686.4 eV. I was measured by the TEM's in–built sensor to be about 2.33 nA, which corresponds to a beam intensity of approximately 30 A/cm^2. By plugging these values into Equation S3 and Equation S4, the estimated temperature increase is just about 0.1522 K which is negligible. Previous TEM experiments also have examined the beam intensity effect on different samples in a similar way, and they all suggest that temperature increase is <1 K[5,10] unless the beam intensity reaches >100 A/cm^2 which was often intentionally used to melt nanostructures.[11] In addition to the theoretical analysis, nanoparticles were never found to show significant morphological change under the electron beam prior to loading, suggesting that the electron beam intensity was within a safe level. For these reasons, electron-beam heating was ignored and the *in situ* testing was considered to occur at room temperature.

S1.5. The Effect of the Electron Beam on Coated Nanoparticles

Silica is known to be more beam sensitive, so the coated nanoparticles were exposed to the electron beam for sufficient time to eliminate any transient behavior. In some cases, the silica overlayer was modified by the electron beam, as shown in Figure S3a-b. In these cases, the thickness decreased somewhat, but stabilized at around 1–2 nm. There was never any change in the metal nanoparticle during this stabilization period. The particle was tested in its stabilized condition.

The present testing of a "coated nanoparticle" can be compared to a prior investigation examining a gold nanoparticle encapsulated by a thick graphitic shell.[12] In that investigation, a highly intense electron beam could be used to shrink the graphitic cage and actually compress the nanoparticle until the material diffused out of a hole in the shell. This is markedly different from the present behavior of a platinum nanoparticle with a silica shell. In that prior work, the graphitic cage is extremely robust, and a high–intensity beam causes it to shrink, but not decompose. In contrast, in Figure S3c-d, a high–intensity beam causes the silica coating to decay until it is almost completely removed. Therefore, the silica overlayer in the present testing under low intensity beam after stabilization serves to reduce surface diffusion, but is not expected to modify the stress state of the particle.

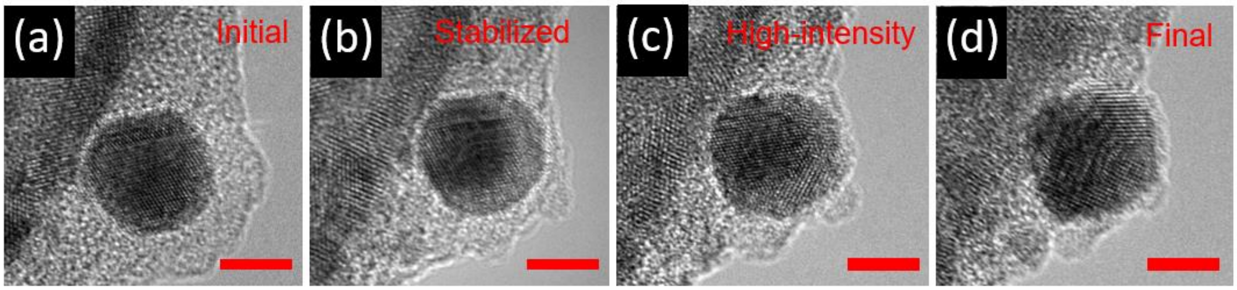

Figure S3: The coated nanoparticle changes slightly but stabilizes under the electron beam. (a) Initially, the coated nanoparticle had a layer thickness of >3 nm. (b) Under the typical-intensity electron beam (30 A/cm^2), the layer thickness gradually decreased and stabilized at 1–2 nm. (c) One coated particle was used to study any effect of coating change on the metal particle. Here, the beam intensity was intentionally increased by focusing the beam (>100 A/cm^2), the layer decreased further. (d) Finally, the layer can be almost removed entirely, and there is some structural change in the underlying metal nanoparticle. All scalebars represent 5 nm.

S2. More Details Regarding Mechanical Behavior of All Nanoparticles

S2.1. Raw Data

The example nanoparticles from the three groups given in Figure 1a–c have typical mechanical responses during loading shown in Figure S4. They all undergo elastic deformation initially, when there is little change in morphology. Then, they undergo plastic deformation, where their morphologies change dramatically. The final stage after unloading the indenter demonstrates that they have undergone permanent morphological change.

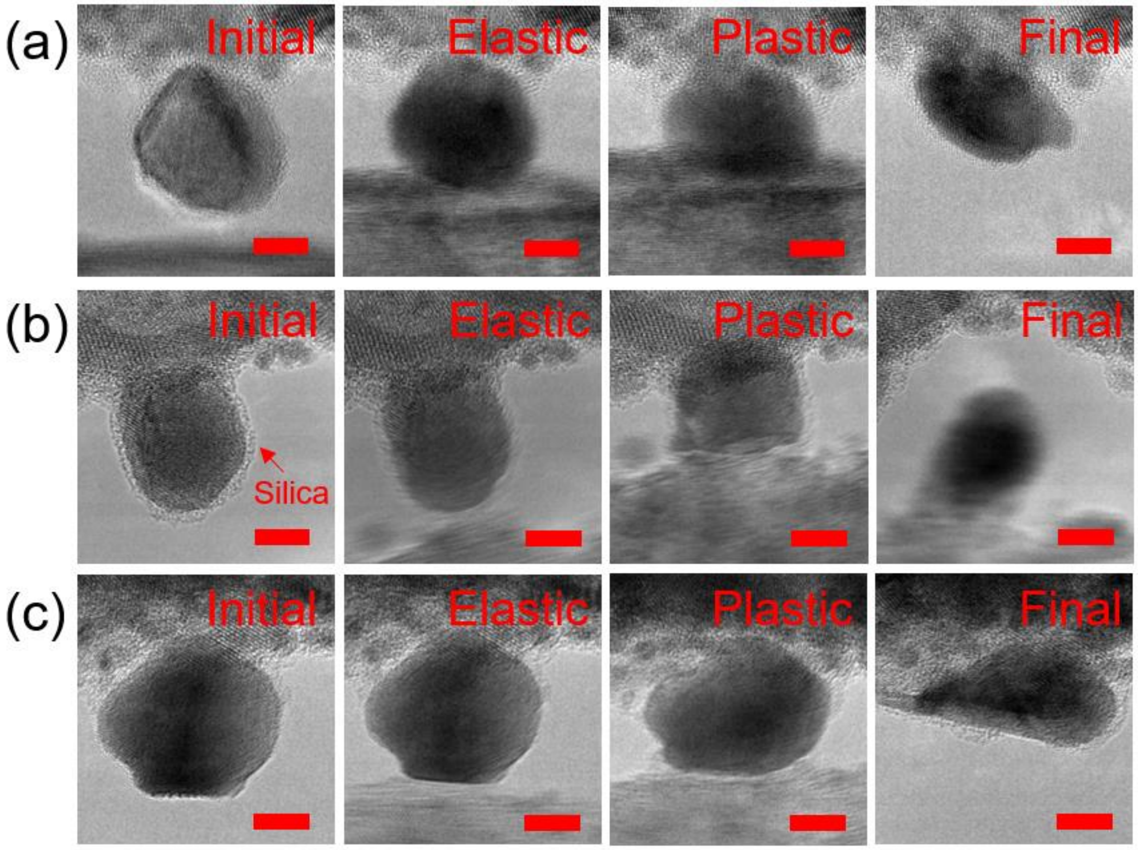

Figure S4: The observed mechanical responses of typical nanoparticles are shown at four stages. Typical nanoparticles from (a) "12–nm bare", (b) "12–nm coated", and (c) "16–nm bare" groups show qualitatively similar mechanical responses. All scalebars represent 5 nm.

The measured yield strengths for all nanoparticles in the three categorized groups are shown in Figure S5. Clearly, within each group, the strength distribution is scattered across a large range without a clear trend, but compared with the two small nanoparticle groups, the average yield strength in the large-nanoparticle group is about 1–2 GPa higher, as shown in Figure S5a. The difference between bare small nanoparticles and coated small nanoparticles is slight. In terms of

elastic strain rate as shown in Figure S5b, there is scatter in the data across an individual group, but without clear trends. The elastic strain rate is in the order of magnitude of 0.01–0.1 s^{-1}.

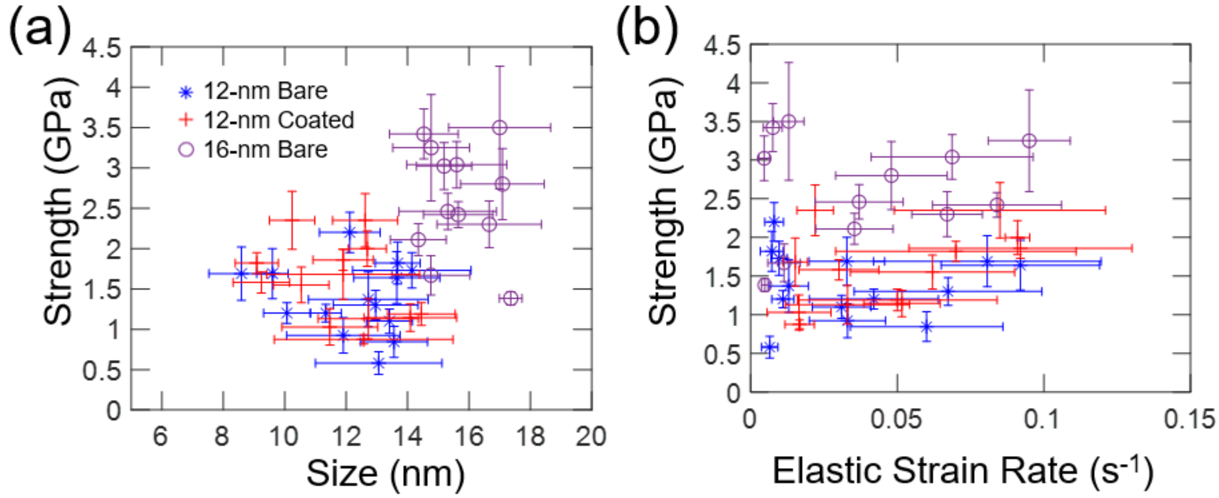

Figure S5: The measured yield strengths for all nanoparticles in the three categorized groups are plotted. The data are shown in terms of (a) nanoparticle size and (b) elastic strain rate. Size uncertainty is indicated by standard deviation, whereas strength and elastic strain rate uncertainty are given by the 95% confidence interval.

S2.2. Loading Direction Effect

The loading direction effect is analyzed by cycling through a large number of loading directions $[ijk]$. Figure 2 confirms the partial-dislocation slip systems of $\{111\}\langle 211\rangle$. The Schmid factor which is defined as $\cos\Phi \cos\lambda$,[13] is calculated by using the preset loading direction $[ijk]$ and accounting for the eight possible (111)-type planes (including both positive and negative cases) with each plane allowing six possible $[211]$-type directions. Hence, in total, each loading direction can have 48 possible values for Schmid factor (not all independent). Figure S6 is the plot for how the maximum and minimum Schmid factor values vary with loading direction. The maximum Schmid factor is of interest, because the material yields first on the slip plane whose Schmid factor is maximum. The upper limit is 0.5 corresponding to the case when Φ=45° and λ=45°, in principle. Though not all directions can reach the condition of upper limit, most of them have Schmid factor close to 0.5. The calculated maximum Schmid factor values have an average of 0.462 with standard deviation of 0.033. The lower limit is just 0.317. Therefore, the loading effect on measured yield

strength is not regarded as a critical effect in this research where nanoparticles were tested with random distribution of loading directions.

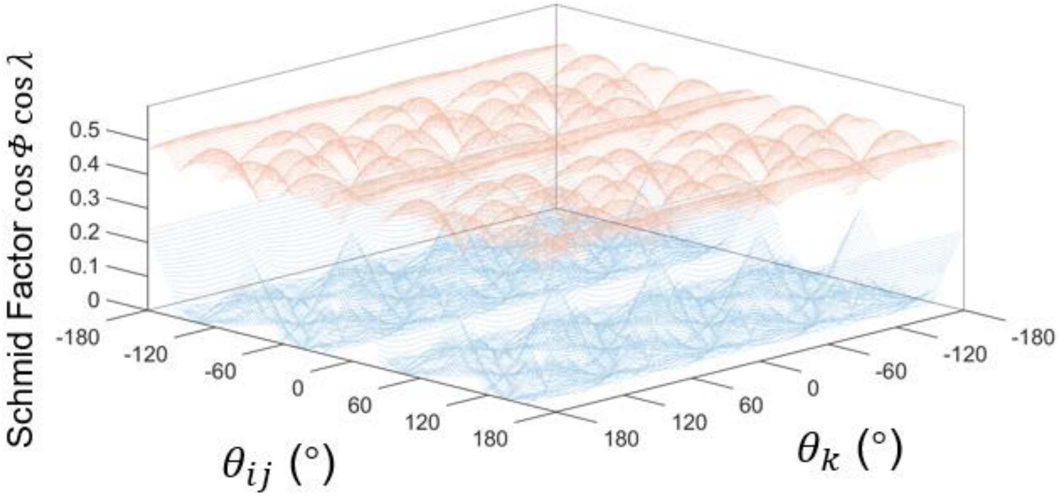

Figure S6: The Schmid Factor (absolute value) varies with loading direction. The loading direction is $[ijk]$. For convenience, loading direction is represented in a spherical coordinate system, where $x_1 = \cos\theta_{ij}\ \sin\theta_k$, $x_2 = \sin\theta_{ij}\ \sin\theta_k$, and $x_3 = \cos\theta_k$. Red data points correspond to maximum possible values, and blue data points correspond to minimum possible values.

Furthermore, Chachamovitz *et al.* pointed out that using either characteristic strength (yield strength or critical resolved shear strength) would not significantly affect the calculation of activation parameters, except for a linear scaling of activation volume as well as athermal strength (See following section for more information).[14] By following their approach, below is the brief derivation paraphrased from their derivations[14] to demonstrate that. If the critical resolved shear stress is simply assumed to scale with yield strength as $\tau_c = m\sigma_y$ by a constant m (Schmid factor, m <1), the cumulative distribution function would be $F(\tau_c) = mF(\sigma_y)$ as well as the distribution width $\omega(\tau_c) = m\omega(\sigma_y)$. Since the activation volume is inversely related to distribution width, the activation volume just scales by the constant: $\Omega(\tau_c) = \Omega(\sigma_y)/m$, and for activation energy, $\Delta G(\tau_c) = \int \Omega(\tau_c)\mathrm{d}\tau_c = \int [\Omega(\sigma_y)/m] m\mathrm{d}\sigma_y = \int \Omega(\sigma_y)\mathrm{d}\sigma_y = \Delta G(\sigma_y)$.[14] In other words, while the absolute values of activation parameters would be expected to vary between a yield-strength analysis and a critical-resolved-shear-strength analysis, the trends between the groups would not be expected to change. Therefore, in this paper, the yield strength of nanoparticles is used as it is

directly measurable by experiment, so that the calculated activation volume as well as athermal strength refer to that of yield strength.

S3. The Detailed Data Analysis from Cumulative Distribution Function

S3.1. Correcting Yield Strength Due to Non–Uniform Distribution of Stress

For a nanoparticle, the effect of a non–uniform distribution of stress on yield strength was previously studied by Mordehai *et al*.[15] Here, we followed their approach. Below is a brief summary of the derivations paraphrased from their work.[15] The nanoparticle's geometry typically has the highest stress around the edges of contact, and the stress drops toward the center of the nanoparticle. A dislocation usually nucleates from the highest–stress edges and propagates to the low–stress center. The resolved shear stress τ has an empirical distribution along this path:[16]

$$\tau(x) = K\sigma\left(\frac{0.5D}{x}\right)^{n} \tag{S5}$$

where σ is the applied normal compressive stress, D is the size of the nanoparticle ($0.5D$ is the radius), x is the distance from the highest–stress surface position, n is stress-decay exponent, and K is a dimensionless parameter. Note that the stress cannot be infinite, and this equation is just approximate to model the stress change along the path of dislocation motion. The initial embryonic dislocation can spontaneously expand the loop from the highest stress position into a loop of characteristic radius x, requiring a negative work $W = -2\pi\int_0^x \tau(x')bx'\mathrm{d}x'$ as well as adding a dislocation line energy E_{dis}:[15]

$$E_{\mathrm{tot}}(x) = W + E_{\mathrm{dis}} = -\frac{2\pi Kb\sigma}{2-n}\frac{(0.5D)^{n}}{x^{n-2}} + \frac{1}{2}Gb^{2}x\ln\left(\frac{2x}{x_0}\right) \tag{S6}$$

where b is Burgers vector, G is the shear modulus, and x_0 is the dislocation's initial core size. The critical dislocation loop size x_{c} and critical compressive stress σ_{c} must meet the condition of $\mathrm{d}E_{\mathrm{tot}}/\mathrm{d}x = 0$ as well as the condition of $E_{\mathrm{tot}}(x_{\mathrm{c}}) = 0$ as the reference energy.[17] The σ_{c} can be finally solved as a result:[15]

$$\sigma_{\mathrm{c}} = \left(\frac{2-n}{1-n}\right)\left(\frac{G}{2^{n+1}\pi eK}\right)\left(\frac{b}{x_0}\right)\left(\frac{x_0}{0.5D}\right)^{n} \propto \left(\frac{x_0}{0.5D}\right)^{n} \tag{S7}$$

This analysis suggests that there is a smaller–is–stronger trend in critical compressive stress just due to the non–uniform stress distribution of the nanoparticle's geometry. This effect on yield strength measurement does not belong to the effect of intrinsic material properties associated with thermal activation, so that it should be ruled out. For our measured raw yield strength data, we corrected them by:

$$\sigma_{\mathrm{y,corrected}} = \sigma_{\mathrm{y}} \left(\frac{D}{D_{\mathrm{avg}}}\right)^{n} \quad \text{(S8)}$$

where the corrected yield strength is normalized to the average size of all measured nanoparticles D_{avg}. n is in the range of 0.5–1, and $n = 0.75$ is selected for typical metal nanoparticles.[15,18,19] Figure S7 shows the raw data (as measured) for reference which can be compared with the stress-concentration-corrected yield strengths of all particles in Figure 3. In this way, the non–uniform stress distribution effect is accounted for, ensuring that this effect does not confound the measured values.

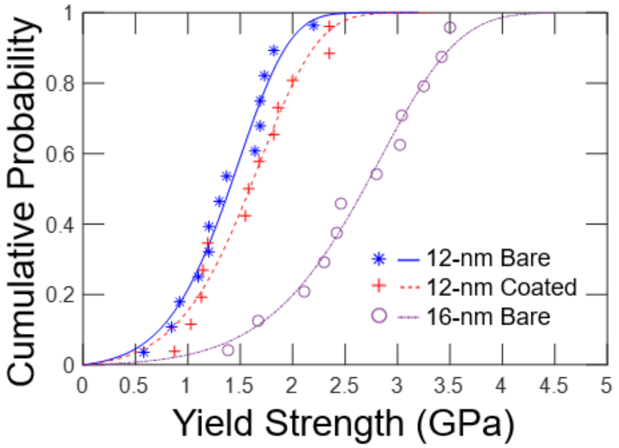

Figure S7: The raw data of measured uncorrected yield strengths can be compared with the corrected yield strengths in Figure 3.

For small nanoparticles, $\sigma_{\mathrm{y,corrected}}$ is lower than measured nominal yield strength, since the non–uniform stress distribution effect increases strength of the small nanoparticles. For large nanoparticles, $\sigma_{\mathrm{y,corrected}}$ is greater, since the non–uniform stress distribution effect reduces the strength of the large nanoparticles. Table S2 shows the calculated activation parameters if the raw data is considered (See following section for more information). Overall, the results do not influence the conclusions of this investigation.

Table S2: Activation parameters are extracted by fitting the cumulative distribution function of measured uncorrected raw yield strength ($\alpha = 4$). Uncertainty is represented as described in Table 1 in the main text.

α=4	Most Probable Strength $\bar{\sigma}$ (GPa)	Activation Volume $\Omega(\bar{\sigma})$ (b^3)	Athermal Strength σ_{ath} (GPa)	Athermal Activation Energy ΔU_{ath} (eV)	Prefactor $N\nu_0$ (s^{-1})
12-nm Bare	1.38±0.01	0.467±0.010	7.28-13.57	0.226-0.309	46.4-1232
12-nm Coated	1.57±0.01	0.403±0.008	7.59-12.89	0.217-0.271	39.1-423.3
16-nm Bare	2.65±0.01	0.302±0.008	13.93-22.60	0.281-0.352	98.4-1894

S3.2. Determining Activation Volume & Most Probable Strength

The determination of activation volume and most probable strength follows the approach of Chachamovitz *et al.*,[14] and below is a brief summary of the paraphrased derivations. The most probable strength $\bar{\sigma}$ appearing at the condition $\partial^2 F(\sigma)/\partial\sigma^2 = 0$ combined with Equation 6, namely, $F(\sigma, T) = 1 - \exp\left[-\frac{N\nu_0}{E\dot{\varepsilon}_{\mathrm{el}}}\int_0^{\sigma}\exp\left(-\frac{\Delta G(\sigma,T)}{k_{\mathrm{B}}T}\right)\mathrm{d}\sigma'\right]$, yields the following relation:[14]

$$\frac{N\nu_0}{E\dot{\varepsilon}_{\mathrm{el}}}\exp\left(-\frac{\Delta G(\bar{\sigma},T)}{k_{\mathrm{B}}T}\right) = \frac{\Omega(\bar{\sigma},T)}{k_{\mathrm{B}}T} \tag{S9}$$

$\Omega(\bar{\sigma}, T)$ may be obtained by fitting experimental data, but a lot of uncertainties are generated as the preset function $\Delta G(\bar{\sigma}, T)$ must be assumed. To simplify the derivation, Chachamovitz *et al.* proposed to introduce a new function through Taylor expansion to second order around $\bar{\sigma}$ defined as:[14]

$$H(\sigma,T) = -\frac{N\nu_0}{E\dot{\varepsilon}_{\mathrm{el}}}\int_0^{\sigma}\exp\left(-\frac{\Delta G(\sigma,T)}{k_{\mathrm{B}}T}\right)\mathrm{d}\sigma' - \frac{\Delta G(\sigma,T)}{k_{\mathrm{B}}T}$$

$$\approx H(\bar{\sigma},T) + \frac{1}{2}\left[-\left(\frac{\Omega(\bar{\sigma},T)}{k_{\mathrm{B}}T}\right)^2 + \frac{\partial\Omega(\bar{\sigma},T)/\partial\sigma}{k_{\mathrm{B}}T}\right](\sigma-\bar{\sigma})^2 \tag{S10}$$

The first derivative $\partial F(\sigma, T)/\partial\sigma$ with the assistance of function $H(\sigma, T)$ can be written as:[14]

$$\frac{\partial F(\sigma,T)}{\partial\sigma} = \frac{N\nu_0}{E\dot{\varepsilon}_{\mathrm{el}}}\exp\big(H(\sigma,T)\big) = \frac{N\nu_0}{E\dot{\varepsilon}_{\mathrm{el}}}\exp\big(H(\bar{\sigma},T)\big)\exp\left(\frac{(\sigma-\bar{\sigma})^2}{2\omega^2}\right) \tag{S11}$$

where $\omega = \left[\left(\frac{\Omega(\bar{\sigma},T)}{k_{\mathrm{B}}T}\right)^2 - \frac{\partial\Omega(\bar{\sigma},T)/\partial\sigma}{k_{\mathrm{B}}T}\right]^{-1/2}$ that is the form of Equation 3 by assuming the first term is far greater than the negligible second term. Therefore, the cumulative distribution function $F(\sigma)$

can be approximately modeled as the cumulative normal distribution centered at $\bar{\sigma}$ with a standard deviation ω:[14]

$$F(\sigma, T) \approx \frac{1}{2}\left[1 + \mathrm{erf}\left(\frac{\sigma - \bar{\sigma}}{\sqrt{2}\omega}\right)\right] \tag{S12}$$

This equation provides a way to determine the most probable strength and activation volume by fitting experimental data without any prior preset or assumption of the form of $\Delta G(\sigma, T)$.

S3.3. Determining Athermal Strength & Athermal Energy

A custom Matlab program is used to fit the yield strength data in the cumulative distribution function in Equation 7. Briefly, the fitting was performed numerically. The best–fit values were coarsely searched across the logarithmic scale to get the order of magnitude at first, and then, precise determination of best–fit values were determined through fine searching therein. By considering the large uncertainty of a multi-parameter logarithmic fit, the activation parameters were finally expressed using a numerical search for all possible values whose errors (root–mean–square residuals) were 1% larger than that of the best–fit values. The derived equation based on transition state theory involved many physical quantities, and the activation parameters can only be determined if other parameters are given with some constraints. The temperature is room temperature, namely 300 K. The melting temperature of the nanoparticles is determined based on the atomistic structure analysis described in the next section. The values of elastic strain rate use the average values of all measured nanoparticles, namely 0.0353 s^{-1}, 0.0489 s^{-1}, and 0.0397 s^{-1} for "12–nm bare" group, "12–nm coated" group, and "16–nm bare" group, respectively. The values of elastic modulus also use the average values of all measured nanoparticles for more accurate calculation. Each nanoparticle's effective elastic modulus is determined by dividing yield strength by elastic strain. The average value of the measured effective elastic modulus values are 33.40 GPa, 32.33 GPa, and 46.37 GPa for "12–nm bare" group, "12–nm coated" group, and "16–nm bare" group, respectively. The measured values are much less than the value of bulk platinum material (180 GPa), which agrees with prior measurements,[20] and is attributable to the geometry of the nanoparticles and spatial inhomogeneities in stress and strain. Finally, the determined parameters ΔU_{ath} and σ_{ath} also have intrinsic constrictions. The activation parameters ΔU_{ath} and σ_{ath} must meet the requirement of Equation 2 at the state of most probable strength:[14]

$$\frac{\Omega(\bar{\sigma},T)}{1-T/T_{\mathrm{m}}} = \Omega(\bar{\sigma}) = \frac{\alpha \Delta U_{\mathrm{ath}}}{\sigma_{\mathrm{ath}}}\left(1-\frac{\bar{\sigma}}{\sigma_{\mathrm{ath}}}\right)^{\alpha-1} \tag{S13}$$

where $\bar{\sigma}$ and $\Omega(\bar{\sigma},T)$ can be experimentally determined from $F(\sigma,T)$ as described before. Currently, there is no commonly agreed-upon method to estimate α, but simulation studies suggest the range of α within 1.5–4 is appropriate.[14,21,22] In main text, $\alpha = 4$ is selected. We compared the results obtained from different α values in Table 1 ($\alpha = 4$), Table S3 ($\alpha = 1.5$) and Table S4 ($\alpha = 2$). Overall, as α increases, all estimated values of activation parameters increase, but the relative difference among the three groups are unchanged. Namely, the difference between 12–nm and 16–nm nanoparticles is significant, while the difference is small between bare and coated nanoparticles of the same size. To choose between these values we rely on Weinberger *et al.*[23] who pointed out that the estimated athermal strengths should in principle be close to the theoretical ideal strength of FCC platinum (in terms of critical resolved shear stress); the results from $\alpha = 4$ are in closest agreement with that and therefore are used in the main text.

Table S3: If α is set to 1.5, the following activation parameters are extracted by fitting the cumulative distribution function of measured yield strength. Uncertainty is represented as described in Table 1 in the main text.

α=1.5	Most Probable Strength $\bar{\sigma}$ (GPa)	Activation Volume $\Omega(\bar{\sigma})$ (b^3)	Athermal Strength σ_{ath} (GPa)	Athermal Activation Energy ΔU_{ath} (eV)	Prefactor $N\nu_0$ (s^{-1})
12-nm Bare	1.30±0.01	0.487±0.023	2.10-2.71	0.157-0.173	6.7-16.0
12-nm Coated	1.46±0.01	0.435±0.009	2.20-2.71	0.155-0.163	6.9-13.0
16-nm Bare	3.03±0.01	0.262±0.006	4.81-6.20	0.196-0.215	7.1-20.9

Table S4: If α is set to 2, the following activation parameters are extracted by fitting the cumulative distribution function of measured yield strength. Uncertainty is represented as described in Table 1 in the main text.

α=2	Most Probable Strength $\bar{\sigma}$ (GPa)	Activation Volume $\Omega(\bar{\sigma})$ (b^3)	Athermal Strength σ_{ath} (GPa)	Athermal Activation Energy ΔU_{ath} (eV)	Prefactor $N\nu_0$ (s^{-1})
12-nm Bare	1.30±0.01	0.487±0.023	2.83-4.49	0.181-0.218	11.6-68.2
12-nm Coated	1.46±0.01	0.435±0.009	3.11-4.60	0.180-0.207	13.5-56.3
16-nm Bare	3.03±0.01	0.262±0.006	6.79-9.38	0.228-0.257	16.5-74.9

S4. Melting Temperature of Bare and Coated Nanoparticles

As described in the main text, the melting temperature can be computed as a function of size and coating using Equation 10. The term A is the ratio of msd of surface atoms of the nanoparticle $\sigma_s^2(D)$ to that of atoms only in the volume of the nanoparticle $\sigma_v^2(D)$. When the surface of the nanoparticle is fully free, $\sigma_s^2(D) > \sigma_v^2(D)$ and therefore, $A > 1$, and melting temperature of the nanoparticle is lower than that of its corresponding bulk material. The largest value of A can be estimated based on Mott's derived expression[24] for the vibrational entropy of melting under the assumption that vibrational entropy of melting dominates the total entropy of metal crystals:[25–27]

$$A_{\max} = \frac{2S_m(\infty)}{3R} + 1 \tag{S14}$$

where $S_m(\infty)$ is the melting entropy of the bulk material, and R is the ideal gas constant.[25] Simply by taking largest value of A, the melting temperature of bare nanoparticles can be determined. The D_0 in Equation 10 is approximately equal to $6h$ for spherical geometry, where h is the atomic diameter.[25–27] For a bare nanoparticle, $A_{\max}$ can be used as the value of A, while for coated nanoparticle $A < A_{\max}$, since a fraction of surface atoms interact with coating. The A of coated nanoparticles, using the approach of Liang *et al.*, can be written as:[27]

$$A = \frac{\left[(1-B)\sigma_s^2(D) + B\sigma_v^2(D)\right]}{\sigma_v^2(D)} = A_{\max}(1 - B) + B \tag{S15}$$

Where B is the ratio of the number of surface atoms passivated by atoms of the coating layer to the total number of surface atoms. Here, the msd of the passivated surface atoms is assumed to be equal to $\sigma_v^2(D)$; in other words, passivated surface atoms behave similar to atoms in the volume. This is a conservative assumption since the binding energy between surface atoms (platinum) and atoms of the coating material (silica) is comparable with the cohesive energy of the metal atoms. For instance, in one simulation study, the cohesive energy of platinum is about 4.73 eV, while the binding energy of platinum with a quartz substrate is about 3–4 eV.[28] Due to structural incompatibility between platinum atoms and silica compounds, silica compounds can only just effectively passivate a fraction of surface atoms, which indicates $B < 1$. The value of B can be estimated in the following way based on the atomic structure of the nanoparticle and coating material. $B = N_1/N_2$, where N_1 is the number of surface atoms passivated by the atoms of coating layers which is roughly equal to $\pi D^2/s$, where s is the area occupied by one molecule of coating

layer (SiO_2) projected onto nanoparticle's surface, and N_2 is the number of surface atoms of the nanoparticle approximately equal to $(D/h)^3(D_0/D)$.[27] Conservatively, though the silica layer shown in the high-resolution TEM image suggests the structure is amorphous, here s is estimated by using the crystalline structure of silica. The determined melting temperatures of bare and coated nanoparticles following this approach are exhibited in Figure S8.

In addition to the thermal stability of the nanoparticle, the thermal stability of the coating material itself is also important. This is because the prerequisite to stabilize a nanoparticle by surface coating is that the coating material itself should be thermally stable. Hence, the melting temperature of the coating layer (silica) is also estimated following the same approach. The difference is that the coating layer takes a thin-film geometry rather than a sphere (layer thickness $D_0 = 2h$).[25–27] Plus, for the coating layer, atoms on one side are completely passivated by platinum atoms, while atoms on the other side are free. This means $B = 0.5$, and $A = (A_{max} + 1)/2$. The determined melting temperatures of the layers at a variety of thicknesses are also added in Figure S8. In this experiment, the thickness of layers is about 1–3 nm, and the corresponding melting temperature is approximately the same as that of the nanoparticles.

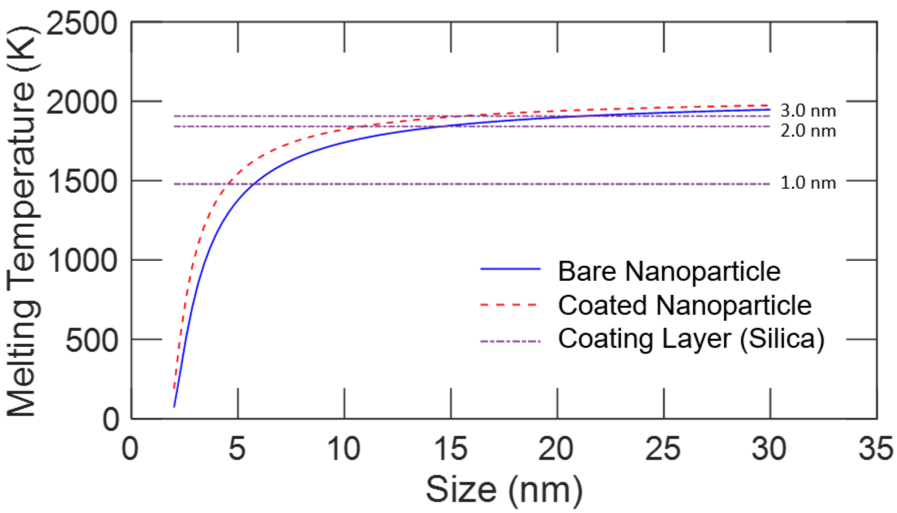

Figure S8: The melting temperature of nanoparticles is related to nanoparticle size and surface conditions. For bare and coated platinum nanoparticles, the parameter values used are as follows: $T_m(\infty)$=2041.4 K,[29] $S_m(\infty)$=10.4 J mol^{-1} K^{-1},[29] h=0.267 nm,[30] and $s \sim 3 \times \sqrt{3} \times l^2$, where l is the Si–O bond length about 0.157 nm for crystalline silica.[31] For the silica coating layer, $T_m(\infty)$=1999 K,[32] $S_m(\infty)$=4.46 J mol^{-1} K^{-1},[32] h=2l, and the calculated melting temperatures for layer thickness of 1 nm, 2 nm, and 3 nm are added for comparison.

References

(1) Sader, J. E.; Chon, J. W. M.; Mulvaney, P. Calibration of Rectangular Atomic Force Microscope Cantilevers. *Rev. Sci. Instrum.* **1999**, *70* (10), 3967–3969. https://doi.org/10.1063/1.1150021.

(2) Sader, J. E.; Sanelli, J. A.; Adamson, B. D.; Monty, J. P.; Wei, X.; Crawford, S. A.; Friend, J. R.; Marusic, I.; Mulvaney, P.; Bieske, E. J. Spring Constant Calibration of Atomic Force Microscope Cantilevers of Arbitrary Shape. *Rev. Sci. Instrum.* **2012**, *83* (10), 103705. https://doi.org/10.1063/1.4757398.

(3) van Eysden, C. A.; Sader, J. E. Frequency Response of Cantilever Beams Immersed in Viscous Fluids with Applications to the Atomic Force Microscope. *Reson. MEMS Princ. Model. Implementation, Appl.* **2015**, *64* (March 1998), 29–53. https://doi.org/10.1002/9783527676330.ch2.

(4) Ding, R.; Espinosa, I. M. P.; Loevlie, D.; Azadehranjbar, S.; Baker, A. J.; Mpourmpakis, G.; Martini, A.; Jacobs, T. D. B. Size-Dependent Shape Distributions of Platinum Nanoparticles. *Nanoscale Adv.* **2022**, *4* (18), 3978–3986. https://doi.org/10.1039/d2na00326k.

(5) Sun, J.; He, L.; Lo, Y.-C.; Xu, T.; Bi, H.; Sun, L.; Zhang, Z.; Mao, S. X.; Li, J. Liquid-like Pseudoelasticity of Sub-10-Nm Crystalline Silver Particles. *Nat. Mater.* **2014**, *13* (11), 1007–1012. https://doi.org/10.1038/nmat4105.

(6) Fisher, S. B. On the Temperature Rise in Electron Irradiated Foils. *Radiat. Eff.* **1970**, *5* (2), 239–243.

(7) Jenčič, I.; Bench, M. W.; Robertson, I. M.; Kirk, M. A. Electron-Beam-Induced Crystallization of Isolated Amorphous Regions in Si, Ge, GaP, and GaAs. *J. Appl. Phys.* **1995**, *78* (2), 974–982. https://doi.org/10.1063/1.360764.

(8) Terada, Y.; Ohkubo, K.; Mohri, T. Thermal Conductivities of Platinum Alloys at High Temperatures. *Platin. Met. Rev.* **2005**, *49* (1), 21–26. https://doi.org/10.1595/147106705X24364.

(9) The Physics Factbook: Density of Platinum

https://hypertextbook.com/facts/2004/OliviaTai.shtml (accessed 2024 -01 -20).

(10) Zheng, H.; Liu, Y.; Cao, F.; Wu, S.; Jia, S.; Cao, A.; Zhao, D.; Wang, J. Electron Beam-Assisted Healing of Nanopores in Magnesium Alloys. *Sci. Rep.* **2013**, *3*, 1–5. https://doi.org/10.1038/srep01920.

(11) Rodrigues, V.; Fuhrer, T.; Ugarte, D. Signature of Atomic Structure in the Quantum Conductance of Gold Nanowires. *Phys. Rev. Lett.* **2000**, *85* (19), 4124–4127. https://doi.org/10.1103/PhysRevLett.85.4124.

(12) Sun, L.; Krasheninnikov, A. V.; Ahlgren, T.; Nordlund, K.; Banhart, F. Plastic Deformation of Single Nanometer-Sized Crystals. *Phys. Rev. Lett.* **2008**, *101* (15), 1–4. https://doi.org/10.1103/PhysRevLett.101.156101.

(13) Schmid, E.; Boas, W. *Plasticity of Crystals with Special Reference to Metals*; F.A.Hughes, 1950.

(14) Chachamovitz, D.; Mordehai, D. The Stress-Dependent Activation Parameters for Dislocation Nucleation in Molybdenum Nanoparticles. *Sci. Rep.* **2018**, *8* (1), 3915. https://doi.org/10.1038/s41598-018-21868-y.

(15) Mordehai, D.; Lee, S. W.; Backes, B.; Srolovitz, D. J.; Nix, W. D.; Rabkin, E. Size Effect in Compression of Single-Crystal Gold Microparticles. *Acta Mater.* **2011**, *59* (13), 5202–5215. https://doi.org/10.1016/j.actamat.2011.04.057.

(16) Paggi, M.; Carpinteri, A. On the Stress Singularities at Multimaterial Interfaces and Related Analogies with Fluid Dynamics and Diffusion. *Appl. Mech. Rev.* **2008**, *61* (1–6), 020801. https://doi.org/10.1115/1.2885134.

(17) Hull, D.; Bacon, D. J. *Introduction to Dislocations*, 4th ed.; Oxford: Butterworth-Heinemann, 2001.

(18) Sharma, A.; Hickman, J.; Gazit, N.; Rabkin, E.; Mishin, Y. Nickel Nanoparticles Set a New Record of Strength. *Nat. Commun.* **2018**, *9* (1), 4102. https://doi.org/10.1038/s41467-018-06575-6.

(19) Zimmerman, J.; Bisht, A.; Mishin, Y.; Rabkin, E. Size and Shape Effects on the Strength

of Platinum Nanoparticles. *J. Mater. Sci.* **2021**, *56*, 18300–18312. https://doi.org/10.1007/s10853-021-06435-7.

(20) Padilla Espinosa, I. M.; Jacobs, T. D. B.; Martini, A. Atomistic Simulations of the Elastic Compression of Platinum Nanoparticles. *Nanoscale Res. Lett.* **2022**, *17* (1), 96. https://doi.org/10.1186/s11671-022-03734-z.

(21) Zhu, T.; Li, J.; Samanta, A.; Leach, A.; Gall, K. Temperature and Strain-Rate Dependence of Surface Dislocation Nucleation. *Phys. Rev. Lett.* **2008**, *100* (2), 025502. https://doi.org/10.1103/PhysRevLett.100.025502.

(22) Chen, L. Y.; He, M. R.; Shin, J.; Richter, G.; Gianola, D. S. Measuring Surface Dislocation Nucleation in Defect-Scarce Nanostructures. *Nat. Mater.* **2015**, *14* (7), 707–713. https://doi.org/10.1038/nmat4288.

(23) Weinberger, C. R.; Jennings, A. T.; Kang, K.; Greer, J. R. Atomistic Simulations and Continuum Modeling of Dislocation Nucleation and Strength in Gold Nanowires. *J. Mech. Phys. Solids* **2012**, *60* (1), 84–103. https://doi.org/10.1016/j.jmps.2011.09.010.

(24) Mott, N. F. The Resistance of Liquid Metals. *Proc. R. Soc. London. Ser. A, Contain. Pap. a Math. Phys. Character* **1934**, *146* (857), 465–472. https://doi.org/10.1098/rspa.1934.0166.

(25) Jiang, Q.; Shi, H. X.; Zhao, M. Melting Thermodynamics of Organic Nanocrystals. *J. Chem. Phys.* **1999**, *111* (5), 2176–2179. https://doi.org/10.30970/jps.22.2601.

(26) Jiang, Q.; Tong, H. Y.; Hsu, D. T.; Okuyama, K.; Shi, F. G. Thermal Stability of Crystalline Thin Films. *Thin Solid Films* **1998**, *312* (1–2), 357–361. https://doi.org/10.1016/S0040-6090(97)00732-3.

(27) Liang, L. H.; Shen, C. M.; Du, S. X.; Liu, W. M.; Xie, X. C.; Gao, H. J. Increase in Thermal Stability Induced by Organic Coatings on Nanoparticles. *Phys. Rev. B - Condens. Matter Mater. Phys.* **2004**, *70* (20), 205419. https://doi.org/10.1103/PhysRevB.70.205419.

(28) Plessow, P. N.; Sánchez-Carrera, R. S.; Li, L.; Rieger, M.; Sauer, S.; Schaefer, A.; Abild-Pedersen, F. Modeling the Interface of Platinum and α-Quartz(001): Implications for Sintering. *J. Phys. Chem. C* **2016**, *120* (19), 10340–10350.

https://doi.org/10.1021/acs.jpcc.6b01403.

(29) Platinum - Specific Heat, Latent Heat of Fusion, Latent Heat of Vaporization https://www.nuclear-power.com/platinum-specific-heat-latent-heat-vaporization-fusion/ (accessed 2022 -12 -25).

(30) Melník, M.; Mikuš, P.; Holloway, C. E. Crystallographic and Structural Characterization of Heterometallic Platinum Clusters Part VIII. Heteronona- and Heterodecanuclear Clusters. *Open Chem.* **2015**, *13* (1), 425–443. https://doi.org/10.1515/chem-2015-0053.

(31) Baur, W. H. The Prediction of Bond Length Variations in Silicon-Oxygen Bonds. *Am. Mineral.* **1971**, *56*, 1573–1599.

(32) Richet, P.; Bottinga, Y. Thermochemical Properties of Silicate Glasses and Liquids: A Review. *Rev. Geophys.* **1986**, *24* (1), 1–25.
